# Editorial: Improving How Orthopaedic Journals Report Research Outcomes Based on Sex and Gender

**DOI:** 10.1097/CORR.0000000000003140

**Published:** 2024-06-10

**Authors:** Seth S. Leopold, Robert N. Hensinger, Andrew J. Schoenfeld, Marc Swiontkowski, Michael J. Rossi, Kimberly J. Templeton

**Affiliations:** 1 Editor-in-Chief, *Clinical Orthopaedics* and *Related Research®,* Schaumburg, IL, USA; 2 Editor-in-Chief, *Journal of Pediatric Orthopaedics*, Ann Arbor, MI, USA; 3 Editor-in-Chief, *Spine*, Boston, MA, USA; 4 Editor-in-Chief, *The Journal of Bone and Joint Surgery*, Needham, MA, USA; 5 Assistant Editor-in-Chief, *Arthroscopy: The Journal of Arthroscopic & Related Surgery*, Wenatchee, WA, USA; 6 Associate Editor, *JBJS Case Connector*, Needham, MA, USA; 7 Past President, Ruth Jackson Orthopaedic Society, Schaumburg, IL, USA; 8 Past President, American Medical Women’s Association, Philadelphia, PA, USA

Sex-based differences in cell biology, tissue function, and anatomy impact disease risk, presentation, and treatment outcomes [12], including in musculoskeletal care [14, 16, 19]. As such, these differences should influence how orthopaedic surgeons and other healthcare professionals conduct research and provide care for patients who have musculoskeletal disease and injury. In addition, gender roles influence interactions with people who conduct research and with healthcare professionals as well as the likelihood that patients will seek care and how they will respond to treatment [7, 8, 12].

Musculoskeletal research, similar to research in other areas of healthcare, does not always disaggregate results based on a patient’s sex or gender [6]. Although some orthopaedic surgery journals have explicit editorial standards on the topic of sex and gender in scientific reporting, and although international entities have published sensible guidelines about it [5], we have observed that these standards are inconsistently applied [6].

Inattention to high-quality standards of scientific reporting can harm patients [13, 20]. Women have been underrepresented in medical research [18], and this trend continues to varying degrees even today, despite mandates to remedy this disparity, at least in federally funded research [2, 15]. However, these mandates include no guidance about how data should be analyzed or reported, thereby limiting the impact of including more women in clinical studies. The care of women has been substantially compromised as a result [1, 3, 10, 17, 21]; not getting this right has sometimes harmed men with certain diagnoses as well [4]. As such, it is no stretch to say that doing better research—and improving how that research is reported in journals—would benefit our patients regardless of their sex or gender.

With this background in mind, leaders of the editorial boards of six orthopaedic journals, along with leaders of funding agencies as well as National Institutes of Health officials, met in November 2023 to discuss these issues. Following that meeting, those editors reached out to the Editors-in-Chief of all indexed orthopaedic surgery journals, seeking concurrence on a few key themes pertaining to the reporting of sex and gender in musculoskeletal research.

The editors who are listed in the byline of this editorial as well as those who are included in its group authorship list (as listed at the end of the editorial) have agreed to the following resolutions:

- We endorse and will apply the Sex and Gender Equity in Research (SAGER) guidelines [9], or we have published in-house guidelines that are substantially similar [11].
- We will discuss building systems into our review processes to make it more likely that authors will follow those guidelines, perhaps including (but not necessarily limited to):
  ○ Adding questions about disaggregation of data by sex and/or gender to our article-submission forms; this applies not only to clinical research but also to laboratory research involving animals and research on cell lines derived from animals or humans.
  ○ Adding questions to our peer-review forms, instructing our reviewers (1) to comment on whether the article under evaluation disaggregated data by sex and/or gender when appropriate (both in clinical research as well as in laboratory research involving animals and research on cell lines derived from animals or humans), and, if this was not done, (2) to suggest that the article explain why such disaggregation was not appropriate in the “Limitations” section of the Discussion.
  ○ Commenting on the handling of sex and/or gender in our rejection letters when this topic is relevant to why a paper was rejected.
- We recognize that while sex is generally considered to be biologic and assigned at birth in the overwhelming majority of people, gender is more challenging to address because (1) the concept of gender is a complex social role designation, (2) gender can be fluid, and (3) the methods of most retrospective studies will be insufficient to characterize it in ways that seem important to us now. When this limitation impacts the interpretation and application of a study’s main findings, we will ask authors to justify it in the Methods section (that is, to explain why incomplete characterization of sex and/or gender is not a disqualifying problem) and to discuss it in the “Limitations” section of the Discussion so as to help readers interpret the findings in light of this methodological shortcoming.
- We recommend that, going forward, clinical researchers seek to suitably characterize participants by sex and/or gender, and laboratory scientists (for research involving animals or cell lines or tissues derived from humans or animals) characterize the sex of the animals or the cell line/tissue source(s). In addition, researchers should plan to analyze and report data disaggregated by these factors when appropriate to their work, so that the influence of these important factors can be better ascertained in orthopaedic research.

We hope that by sharing these resolutions with readers, many of whom are also researchers and representatives on institutional review boards (IRBs), institutional animal care and use committees (IACUCs), and/or funding agencies and organizations, the orthopaedic research of the future will be both better designed and better reported.

## Group Authors

The members of the Sex and Gender Research in Orthopaedic Journals Group include: Nicola Maffulli MD, MS, PhD, FRCP, FRCS(Orth), Editor-in-Chief, *Journal of Orthopaedic Research and Surgery* and *Muscles, Ligaments and Tendons Journal;* Jeffrey C. Wang MD, Jens R. Chapman MD, and Karsten Wiechert MD, Editors-in-Chief, *Global Spine Journal*; Steven L. Kates MD, Editor Emeritus, and Simon Mears MD, PhD, Editor-in-Chief, *Geriatric Orthopaedic Surgery & Rehabilitation;* Michael A. Mont MD, Editor-in-Chief, *The Journal of Arthroplasty*; Marius M. Scarlat MD, PhD, Editor-in-Chief, *International Orthopaedics;* Dr. Ashok N. Johari MS, MCh, FCPS, FRCS, FAMS, PhD(Hc), Editor-in-Chief, *Journal of Pediatric Orthopaedics B;* Professor Fares S. Haddad, Editor-in-Chief, *The Bone & Joint Journal;* Frederick M. Azar MD, Editor-in-Chief, *Orthopedic Clinics of North America;* James H. Lubowitz MD, Editor-in-Chief, *Arthroscopy: The Journal of Arthroscopic & Related Surgery;* Peter V. Giannoudis BSc, MD, FACS, FRCS (Glasg), FRCS (Eng), Editor-in-Chief, *Injury: International Journal of the Care of the Injured;* Charles N. Cornell MD, Editor-in-Chief, *HSS Journal: The Musculoskeletal Journal of Hospital for Special Surgery;* Joy C. MacDermid PT, PhD, Editor-in-Chief, *Journal of Hand Therapy;* Jon Karlsson MD, PhD, Editor-in-Chief, *Knee Surgery, Sports Traumatology, Arthroscopy;* Mauro Alini PhD, Robert L. Mauck PhD, and Daisuke Sakai MD, PhD, Editors-in-Chief, *JOR Spine;* David Hunter MBBS (Hons), MSc (Clin Epi), MSpMed, PhD, FRACP (Rheum), and Anne-Marie Malfait MD, PhD, co-Editors-in-Chief, *Osteoarthritis and Cartilage;* Søren Overgaard, Editor-in-Chief, *Acta Orthopaedica*; Henri Migaud MD, Editor-in-Chief, *Orthopaedics & Traumatology: Surgery & Research;* William J. Mallon MD, Editor-in-Chief, *Journal of Shoulder and Elbow Surgery*; Clare L. Ardern PT, PhD, Editor-in-Chief, *Journal of Orthopaedic & Sports Physical Therapy,* Christopher Bono MD, Editor-in-Chief, *The Spine Journal*; and Jefferson Brand MD, Editor-in-Chief, *Sports Medicine and Arthroscopy Review.*
